# Natural Killer Cell (NK-92MI)-Based Therapy for Pulmonary Metastasis of Anaplastic Thyroid Cancer in a Nude Mouse Model

**DOI:** 10.3389/fimmu.2017.00816

**Published:** 2017-07-21

**Authors:** Liya Zhu, Xiu Juan Li, Senthilkumar Kalimuthu, Prakash Gangadaran, Ho Won Lee, Ji Min Oh, Se Hwan Baek, Shin Young Jeong, Sang-Woo Lee, Jaetae Lee, Byeong-Cheol Ahn

**Affiliations:** ^1^Department of Nuclear Medicine, Kyungpook School of Medicine, Kyungpook National University, Kyungpook National University Hospital, Daegu, South Korea; ^2^Department of Radiology, Taian City Central Hospital, Taian, China

**Keywords:** natural killer cells, immunotherapy, anaplastic thyroid cancer, pulmonary metastasis, mouse model

## Abstract

**Objective:**

Natural killer (NK) cells represent the third largest population of lymphocytes, and they play an important role in immune surveillance against tumors. The lungs are a common metastatic site for anaplastic thyroid cancer (ATC), and metastasis is one of the most frequent causes of mortality in this type of cancer. In the current study, we evaluated the effects of NK cell-based immunotherapy for pulmonary metastasis of ATC and determined how it affects the effector molecules of NK cells.

**Methods:**

Human NK cells (NK-92MI) were retrovirally transduced to express the effluc gene. Human ATC cells (CAL-62) were transduced with the effluc and Rluc genes. The cytotoxicity of NK cells against CAL-62 cells was assessed using the CytoTox 96^®^ Non-Radioactive Cytotoxicity Assay system. Pulmonary metastases of ATC were developed by i.v. injection of CAL-62, and metastasis growth was monitored using bioluminescence imaging (BLI). To treat the metastases, five million NK-92MI cells were injected twice into the caudal vein of nude mice. To assess the targetability of NK cells to ATC tumors, NK-92MI cells expressing the effluc gene (NK/F) were administered through the tail vein of nude mice with a pulmonary metastasis or tumor xenograft. BLI was subsequently performed at 1, 3, 24, and 48 h.

**Results:**

NK/F and CAL-62 cells expressing the effluc or Rluc gene (CAL-62/F, CAL-62/R) were successfully established. Expression of the effluc and Rluc genes in NK/F, CAL-62/F, and CAL-62/R cells was verified by RT-polymerase chain reaction, western blotting, and luciferase assay. After coculture of NK-92MI and CAL-62/F cells for 24 h, the BLI signal intensity of CAL-62/F cells proportionally decreased with the number of cocultured NK cells. An ATC pulmonary metastasis mouse model was successfully generated, and NK cells significantly inhibited the growth of the metastasis (*p* < 0.01). The NK/F cells exhibited targetability to the pulmonary metastasis and tumor xenograft in the mouse model.

**Conclusion:**

The results of present study suggest that NK cells are able to target ATC tumors and that NK cell-based immunotherapy may serve as an effective therapeutic approach for pulmonary metastases of ATC.

## Introduction

Although differentiated thyroid cancer constitutes >90% of thyroid cancer cases and has an excellent prognosis, anaplastic thyroid cancer (ATC), an undifferentiated subtype of thyroid cancer, is one of the most fatal malignancies, with a mean survival of 2–6 months and an overall 5-year survival rate of up to 14%. The poor prognosis of ATC is known to be partly due to early metastases of the malignancy, which are not surgically removable and are unresponsive to conventional chemotherapy or radiotherapy (1). Pulmonary metastasis of ATC is usually multifocal; therefore, these metastases are almost always not surgically resectable (2). New effective therapeutic strategies for pulmonary metastasis of ATC might improve the prognosis of this cancer.

The immune system plays a crucial role in combating tumors, and it has promising potential in the oncologic arena. Natural killer (NK) cells are able to kill target cells without prior stimulation, and their use is more feasible compared to T cells, which need prior sensitization. The *in vivo* tumoricidal effect of NK cells on various malignancies, including thyroid cancer, has been reported in previous studies (3–7). Erik Wennerberg et al. reported that human ATC cells are sensitive to NK cell-mediated lysis *via* ULBP2/5/6, and are able to chemoattract NK cells. The cytotoxic mechanism of NK-92MI in ATC has yet to be clearly explored. However, Huang et al. demonstrated that the mechanism is dependent on the expression level of NKG2D ligand on target cancer cells (8). Furthermore, Ksienzyk et al. reported that NK cells can inhibit pulmonary metastasis formation after IFN-γ treatment in a mouse model of colon cancer (9). The lungs are the most common metastatic site of ATC, followed by bone, and the metastases usually are not surgically resectable (2, 10). Therefore, new effective therapeutic strategies for pulmonary metastasis of ATC are urgently needed, and NK cell-based immunotherapy might represent a therapeutic strategy for the metastases. The present study determined whether ATC pulmonary metastases would be a suitable target for NK cell-based immunotherapy.

Non-invasive *in vivo* cell trafficking is an essential tool for developing immune cell-based therapies because it provides information on the biodistribution of therapeutic cells in living subjects. As reported in previous studies, various imaging techniques, such as optical imaging, PET, SPECT, and MRI, have been applied to analyze NK cell trafficking (11–15). For *in vivo* trafficking, therapeutic cells should be visible with the imaging modalities, and direct and indirect labeling methods are generally applied to improve the sensitivity of each imaging modality. For *in vivo* tracking, cells can be directly labeled with signal-emitting agents such as fluorophores, radionuclides, and iron oxides, or indirectly labeled with reporter genes (16–19). Although indirect cell labeling is difficult to accomplish as compared to direct labeling, it has many advantages over direct labeling, such as having no dilution effect and allowing for long-term monitoring (20). Long-term monitoring of NK cells in living animals might provide invaluable information for the development of NK cell-based immunotherapy; however, long-term monitoring of NK cells using reporter gene technology in an animal model with tumors has yet to be reported (15, 21, 22). In the current study, an optical reporter gene was transduced into NK-92MI cells to assess the long-term fate of NK cells *in vivo*.

In addition, we also developed a reliable mouse model of ATC pulmonary metastasis and investigated the effect of NK cell-based immunotherapy on the pulmonary metastases.

## Materials and Methods

### Cell Culture

The human thyroid cancer cell line CAL-62 was purchased from the American Type Culture Collection (Rockville, MD, USA). The thyroid cancer cells were maintained in DMEM/high-glucose medium (HyClone Laboratories, Inc., South Logan, UT, USA) supplemented with 10% FBS and 100 U/ml penicillin/streptomycin. The NK-92MI human NK cell line was obtained from the American Type Culture Collection. NK-92MI cells were incubated in stem cell growth medium (Cellgro, Freiburg, Germany) supplemented with 2% human serum and 100 U/ml penicillin/streptomycin. CAL-62 cells were transduced with lentivirus co-expressing the Rluc (Renilla luciferase), mcherry, and puromycin genes under the control of the CMV promoter (Genecopoeia, Rockville, MD, USA). Stable clones were selected with puromycin (Sigma-Aldrich, St. Louis, MO, USA). CAL-62 and NK-92MI cells were retrovirally transduced to express both the effluc (enhanced firefly luciferase) and Thy1.1 genes. Thy1.1-positive cells were sorted by CD90.1 microbeads (Miltenyi Biotec, Bergisch Gladbach, Germany). The established stable cell lines expressing the Rluc and effluc genes were referred to as the CAL-62/R, CAL-62/F, and NK/F cells.

### RT-Polymerase Chain Reaction (PCR) Analysis for the Rluc and Effluc Genes

CAL-62, CAL-62/R, CAL-62/F, NK-92MI, and NK/F cells were lysed using TRIzol solution (Invitrogen, Carlsbad, CA, USA), and total RNA was extracted according to the manufacturer’s instructions. Reverse transcription was performed using the Revert Aid First Strand cDNA Synthesis kit (Fermentas, ON, Canada). PCR was carried out using i-Taq DNA polymerase (iNtRON Biotechnology, Seongnam, Korea) and a GeneAmp PCR system. The samples were denatured for 2 min at 94°C, followed by 40 cycles of amplification at 94°C for 20 s, 57°C for 10 s, and 72°C for 30 s. This was followed by a final elongation at 72°C for 5 min. The following primers were used: the effluc gene, forward: 5′-GCACAAGGCCATGAAGAGAT-3′, reverse: 5′-CTTCTTGCTCACGAACACCA-3′; the Rluc gene, forward: 5′-TATGATTCCGAGAAGCACGC-3′, reverse: 5′-TGATCCAGGAGGCGATATGA-3′; and *GAPDH*, forward: 5′-AGTGATGGCATGGACTGTGG-3′, reverse: 5′-GTCAAGGCTGAGAACGGGAA-3′. PCR products were separated by electrophoresis in an ethidium bromide-stained agarose gel.

### Western Blotting

Briefly, CAL-62, CAL-62/R, and CAL-62/F cells were plated and allowed to attach for 24 h, and NK/F cells were cultured in 75 cm^2^ flasks. Cells were harvested and suspended in RIPA buffer (Thermo Fisher Scientific, Suwanee, GA, USA). Extracts were incubated on ice for 30 min and centrifuged at 13,200 rpm for 20 min at 4°C. After centrifugation, the supernatants were collected, and the protein concentrations were determined using a BSA protein assay kit (Pierce Biotechnology, Inc., Rockford, IL, USA). Whole lysates were resolved on an SDS-PAGE gel and transferred to PVDF membranes (Bio-Rad, Hercules, CA, USA). Membranes were probed with the specific primary antibodies and then with peroxidase-conjugated secondary antibodies. Bands were visualized with an enhanced chemiluminescence kit (Amersham, Arlington Heights, IL, USA). The following antibodies were used: antibodies against Renilla luciferase, luciferase, and β-actin (Abcam, Promega, Cell Signaling Technology). To analyze the mechanism of the antitumor effects of NK cells, we also checked the expression of the following proteins that function in the apoptosis signaling pathway by western blotting: caspase-3, cleaved-caspase-3, cleaved PARP, cytochrome-C, and β-actin (Cell Signaling Technology).

### Luciferase Assay

To examine luciferase activity *in vitro*, 1.25 × 10^4^, 2.5 × 10^4^, 5 × 10^4^, 1 × 10^5^, and 2 × 10^5^ CAL-62, CAL-62/R, and CAL-62/F cells were plated in black 96-well plates and incubated for 24 h. Coelenterazine or d-luciferin was added to each well, followed by measurement of the bioluminescent signal using an IVIS lumina III imaging system (Caliper, Alameda, CA, USA).

### Cytotoxicity Assay

The cytotoxic activity of NK-92MI cells was assessed using the CytoTox 96^®^ Non-Radioactive Cytotoxicity Assay system (Promega, Madison, WI, USA). A total of 5 × 10^3^ CAL-62/F cells were cultured in a round-bottom, 96-well plate. The effector NK-92MI cells were distributed in triplicate at effector:target (E:T) cell ratios of 0.1:1, 0.5:1, 1:1, 2.5:1, and 5:1. After a 4 h incubation at 37°C in 5% CO_2_, the supernatant in each well was harvested and transferred to a new plate. The fluorescence emitted by the samples was measured using a microplate reader (Bio-Rad, Hercules, CA, USA). Data are expressed as arbitrary fluorescent units. NK cell cytotoxicity was calculated using the following equation:

$$\begin{array}{l} \text{NK cell cytotoxicity}\left(\%\right)\\ =\frac{\text{Experimental}-\text{Effector Spontaneous}-\text{Target Spontaneous}}{\text{Target Maximum}-\text{Target Spontaneous}}\times 100\% \end{array}$$

To confirm that the sensitivity of CAL-62 cells to NK cell cytotoxicity was not modified by transfection, the CCK-8 assay was also performed after the CAL-62 cells have been cocultured with NK cells for 24 h.

### *In Vivo* Animal Experiments

Specific pathogen-free 6-week-old female BALB/c nude mice (Hamamatsu, Shizuoka, Japan) were used for the *in vivo* study. All animal experiment protocols were conducted in accordance with the National Institutes of Health guidelines for the care and use of laboratory animals and approved by the Committee for the Handing and Use of Animals of Kyungpook National University.

### Establishment of Nude Mouse Model of ATC Pulmonary Metastasis

The protocol published by the Varki group was used to establish a pulmonary metastasis animal model (23). Briefly, CAL-62/F cells were grown in complete medium. When the cells were 70% confluent, the medium was replaced with fresh medium to remove dead and detached cells. Subsequently, 10^6^ cells were suspended in 150 µl PBS and injected into mice *via* the tail vein. Immediately following injection, bioluminescence imaging (BLI) imaging was performed with 100 µl of d-luciferin (3 mg/mouse; Caliper). The development of pulmonary metastasis was monitored weekly for 7 weeks using the BLI signal.

### *In Vivo* NK Cell Treatment

Study 1: After the confirmation of pulmonary metastasis with *in vivo* BLI, the mice were randomized into two groups, and received either 5 × 10^6^ NK-92MI cells or PBS through the intravenous route for 2 days. BLI was performed to monitor tumor growth at days 1, 3, and 7 after treatment.

Study 2: The therapeutic effect of multiple injections of NK-92MI was also evaluated with *in vivo* BLI. Second and third injections of NK-92MI were performed when the tumor started to regrow after previous NK treatment, and the BLI was obtained for 2 weeks after the third treatment.

### *Ex Vivo* 

To confirm the correlation between BLI signal intensity and tumor burden, the mice were sacrificed after whole body imaging, and their lungs were isolated and imaged. The lungs were then fixed in formalin and processed into paraffin blocks for hematoxylin and eosin staining.

### NK Cell Tracking

Study 1: An *in vivo* NK cell bio-distribution study was performed with BALB/c female nude mice using the following procedures: CAL-62/R cells (1 × 10^6^ cells/100 μl PBS) were injected into the tail vein or subcutaneous tissue of the right thigh. After 21 or 42 days, NK/F cells suspended in PBS (1 × 10^7^ cells/100 µl) were intravenously injected. BLI was performed using the IVIS Lumina III imaging system at 1, 3, 24, and 48 h after injection of NK/F cells.

Study 2: To investigate how NK cells reach the lung metastases and where they reside during the period of metastasis formation, five million NK/F cells were injected into BALB/c female nude mice after the intravenous injection of CAL-62 cells. To determine how long these NK cells were retained in the metastases, the BLI was obtained until the NK/F cell signal disappeared from the mice.

## Results

### Establishment of Reporter Gene-Expressing Stable Cell Lines

**Figure 1:**
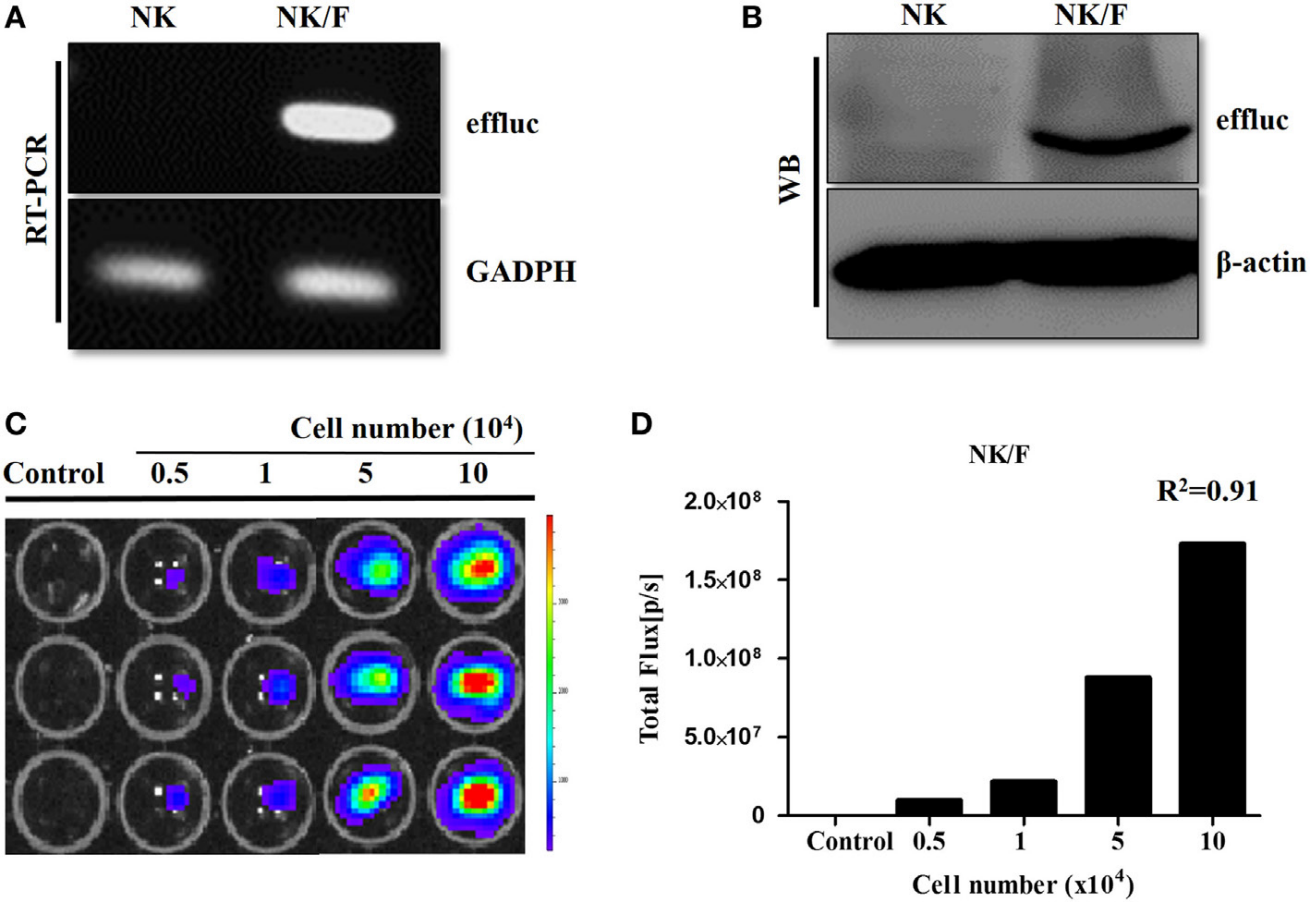
Establishment of NK cells expressing a reporter gene. **(A)** RT-polymerase chain reaction and **(B)** western blotting analyses showing effluc expression at the mRNA and protein levels, respectively, in NK/F cells. **(C,D)** Firefly luciferase activity was determined by bioluminescence imaging, and the intensity of the effluc was correlated with the cell number, *R*^2^ = 0.91. effluc, enhanced firefly luciferase; Rluc, Renilla luciferase; NK/F, NK-92MI/effluc cells.

Anaplastic thyroid cancer cells and human NK cells were transfected with retrovirus containing the effluc gene and Rluc gene to generate cells that expressed a reporter gene. The successful insertion of the effluc gene into NK cells was confirmed by RT-PCR and western blotting (Figures 1A,B). The luciferase activity of NK/F cells revealed a 1,922-fold higher BLI signal compared to that in the parental NK-92MI cells (9 × 10^4^ ± 8.87 × 10^4^ vs 1.73 × 10^8^ ± 6.42 × 10^6^ photon flux) (Figures 1C,D). Furthermore, the correlation of the intensities of the measured BLI signals with the cell line was around 0.91. The reporter gene expression of CAL-62/F and CAL-62/R cells was analyzed by RT-PCR, western blotting, and BLI (Figure S1 in Supplementary Material).

### NK-92MI Cell Cytotoxicity against CAL-62/F Cells

**Figure 2:**
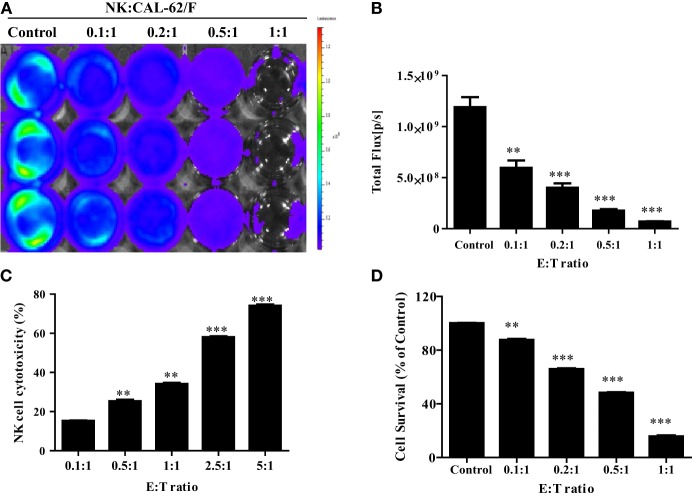
NK cell cytotoxicity to CAL-62/F (CAL-62) cells. Bioluminescence signals were monitored after NK cells were co-incubated with CAL-62/F at E:T ratios of 0.1:1, 0.2:1, 0.5:1, and 1:1 for 24 h. **(A,B)** The intensity of bioluminescence imaging signals in tumor cells was decreased a dose-dependent manner. **(C)** Cytotoxic activity of NK cells by using the CytoTox 96^®^ Non-Radioactive Cytotoxicity Assay system. **(D)** The antitumor effect of die NK cells to the CAL-62 was measured by the CCK-8 assay. Experiments were performed at least in triplicate, and values were plotted as mean ± SE. **p < 0.01, ***p < 0.001. effluc; enhanced firefly luciferase; NK, NK-92MI cells; CAL-62/F, CAL-62/effluc cells.

To demonstrate the *in vitro* cytotoxic effect of NK cells against CAL-62 cells, NK cells were co-incubated with CAL-62/F cells at E:T ratios of 0.1:1, 0.2:1, 0.5:1, and 1:1. The intensity of the BLI signal in tumor cells showed a dose-dependent decrease as the NK cell number increased (Figure 2A). From the quantitative data, the intensity in the BLI signal at a 1:1 ratio of was 15-fold lower compared to the control group (7.04 × 10^7^:1.19 × 10^9^) (Figure 2B). The cytotoxic effect of NK cells on CAL-62 cells *in vitro* was measured using the CytoTox 96^®^ Non-Radioactive Cytotoxicity Assay system and the CCK-8 assay. Both cytotoxic assays showed similar results (Figures 2C,D).

### Establishment of a Nude Mouse Model of ATC Pulmonary Metastasis

**Figure 3:**
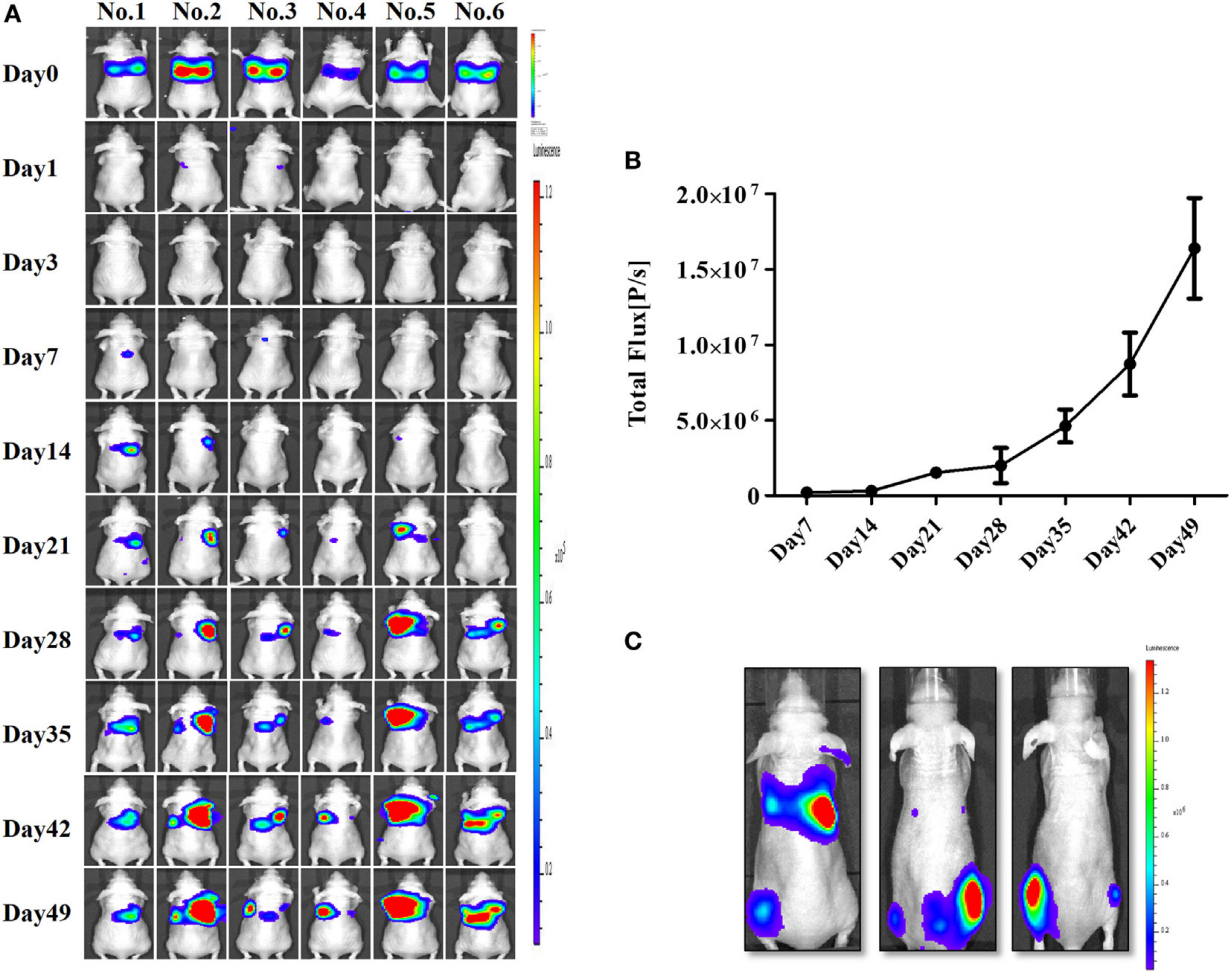
Generation of an anaplastic thyroid cancer pulmonary metastasis mouse model by intravenous injection of CAL-62/F cells. **(A)** Bioluminescence imaging (BLI) of mice after injection of CAL-62/F cells. **(B)** Average BLI signal intensities of the mice. **(C)** Bone metastases developed in some mice (almost 15%) 6 weeks after the injection. effluc, enhanced firefly luciferase; CAL-62/F, CAL-62/effluc cells.

After confirming the presence of strong BLI signals in the CAL-62/F cells *in vitro*, we determined whether we could accurately measure CAL-62/F tumor size using the BLI signal intensity in mouse lungs. CAL-62/F cells were injected *via* the tail vein, and sequential BLI analyses revealed that all mice that received the CAL-62/F cells showed similar tumor development (Figure 3A). BLI signals were detected in the lung area right after injection, and these signals disappeared over time. BLI signals in the lung reappeared at day 7 and increased rapidly (Figure 3B). Six weeks postinjection, BLI signals were detected in the hind limbs of several mice, indicating bone metastasis in the femurs (Figure 3C).

### NK Cell-Based Immunotherapy for Pulmonary Metastasis of ATC

**Figure 4:**
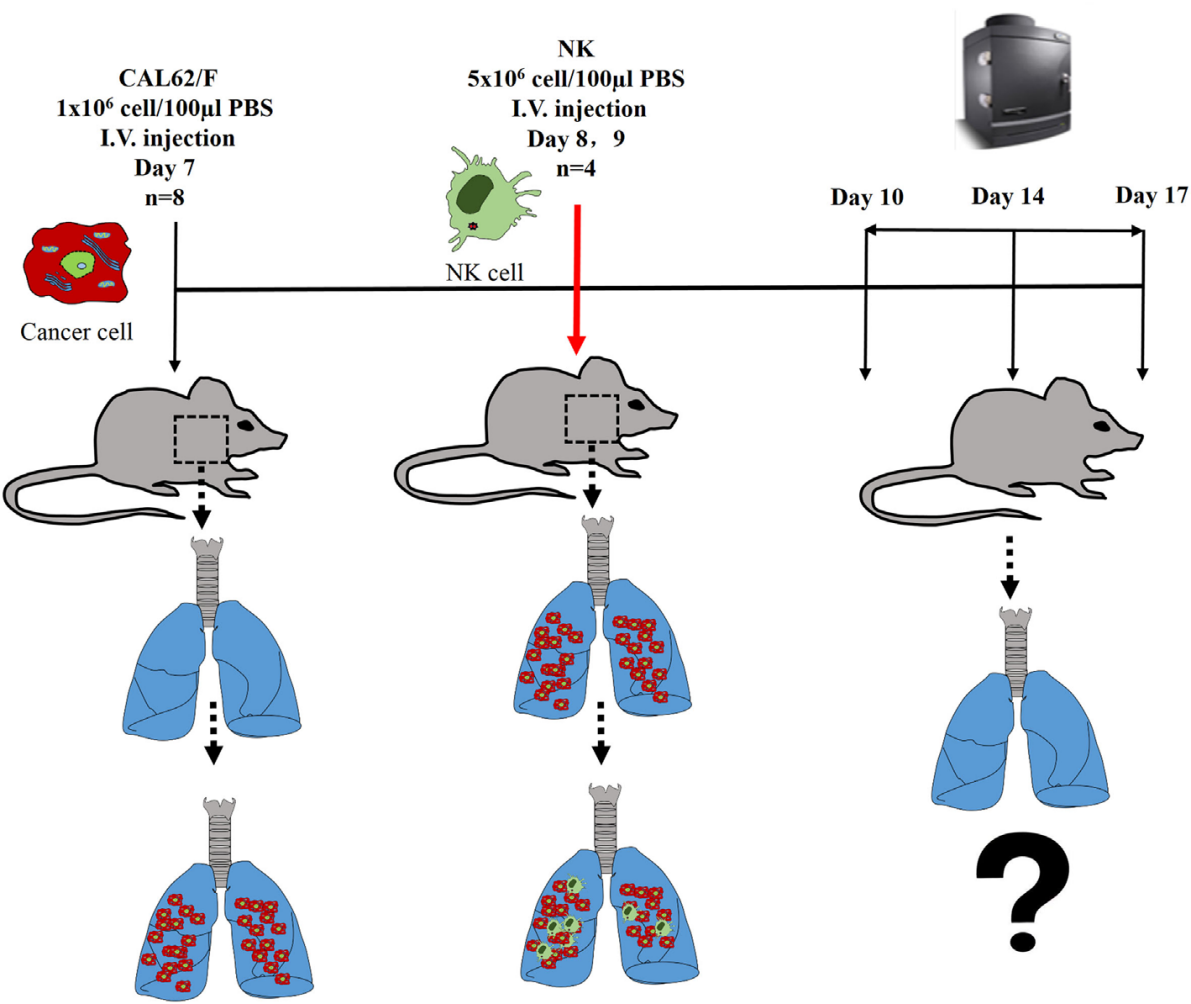
The experimental schema of the *in vivo* NK cell-based immunotherapy for anaplastic thyroid cancer pulmonary metastases.

**Figure 5:**
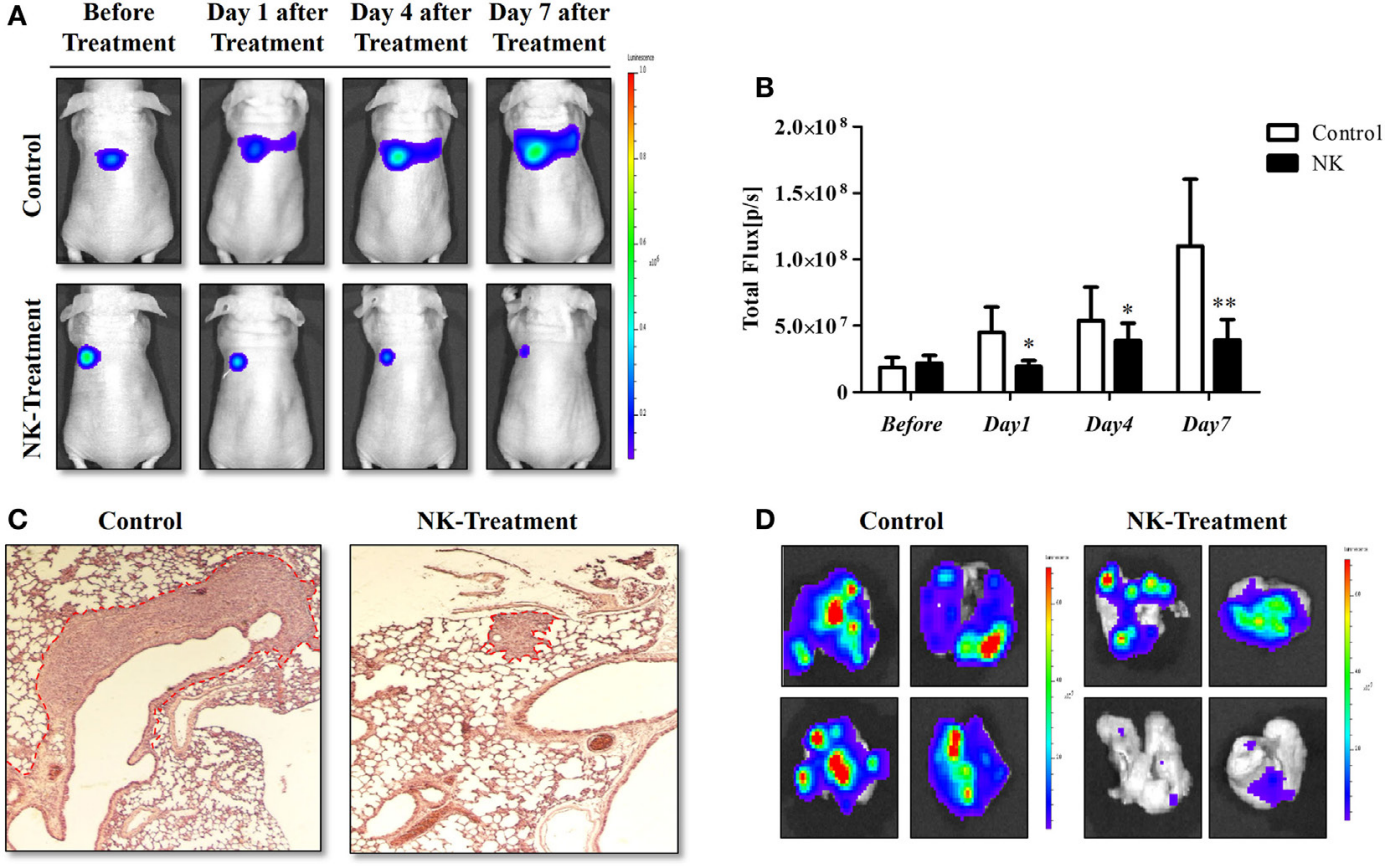
NK cell-based immunotherapy for anaplastic thyroid cancer (ATC) pulmonary metastasis. CAL-62/F cells were injected intravenously *via* the tail vein of nude mice, and after 7 days, NK cells were intravenously injected. After the *in vivo* experiment, the mice were sacrificed, and *ex vivo* experiments were performed. **(A)** *In vivo* bioluminescence imaging (BLI) of an ATC tumor. **(B)** Quantification of the BLI signals. **(C)** Hematoxylin and eosin staining of the ATC pulmonary metastases. **(D)** *Ex vivo* BLI of the ATC pulmonary metastases. Experiments were performed at least in triplicate, and the values represent the mean ± SE. *p < 0.05, **p < 0.01. effluc, enhanced firefly luciferase; CAL-62/F, CAL-62/effluc cells; NK, NK-92MI cells.

The experimental scheme for the *in vivo* NK cell-based immunotherapy for ATC pulmonary metastasis is presented in Figure 4. The control mice showed increased BLI signals over time, indicating growth of the pulmonary metastasis (Figure 5A). On the contrary, mice that received the injection of NK cells exhibited stationary or inhibited growth of the metastasis. Seven days after NK treatment, the BLI signal intensity in the control group was 2.8-fold higher than that in the NK treatment group (*p* < 0.05) (Figure 5B). Tumor size as assessed by histology and *ex vivo* BLI imaging was larger in the control group than in the NK treatment group (Figures 5C,D).

A second study was performed to explore the duration of the therapeutic effect of NK cells and the efficacy of repetitive NK cell therapy. The results of study 2 indicated that the antitumor effect of NK cells lasted for about 10–14 days. In addition, NK cells still showed significant therapeutic effect toward the tumors, and the cancer did not develop resistance against NK cell therapy even after the third injection (Figure S4 in Supplementary Material).

### NK-92MI Cell Trafficking

**Figure 6:**
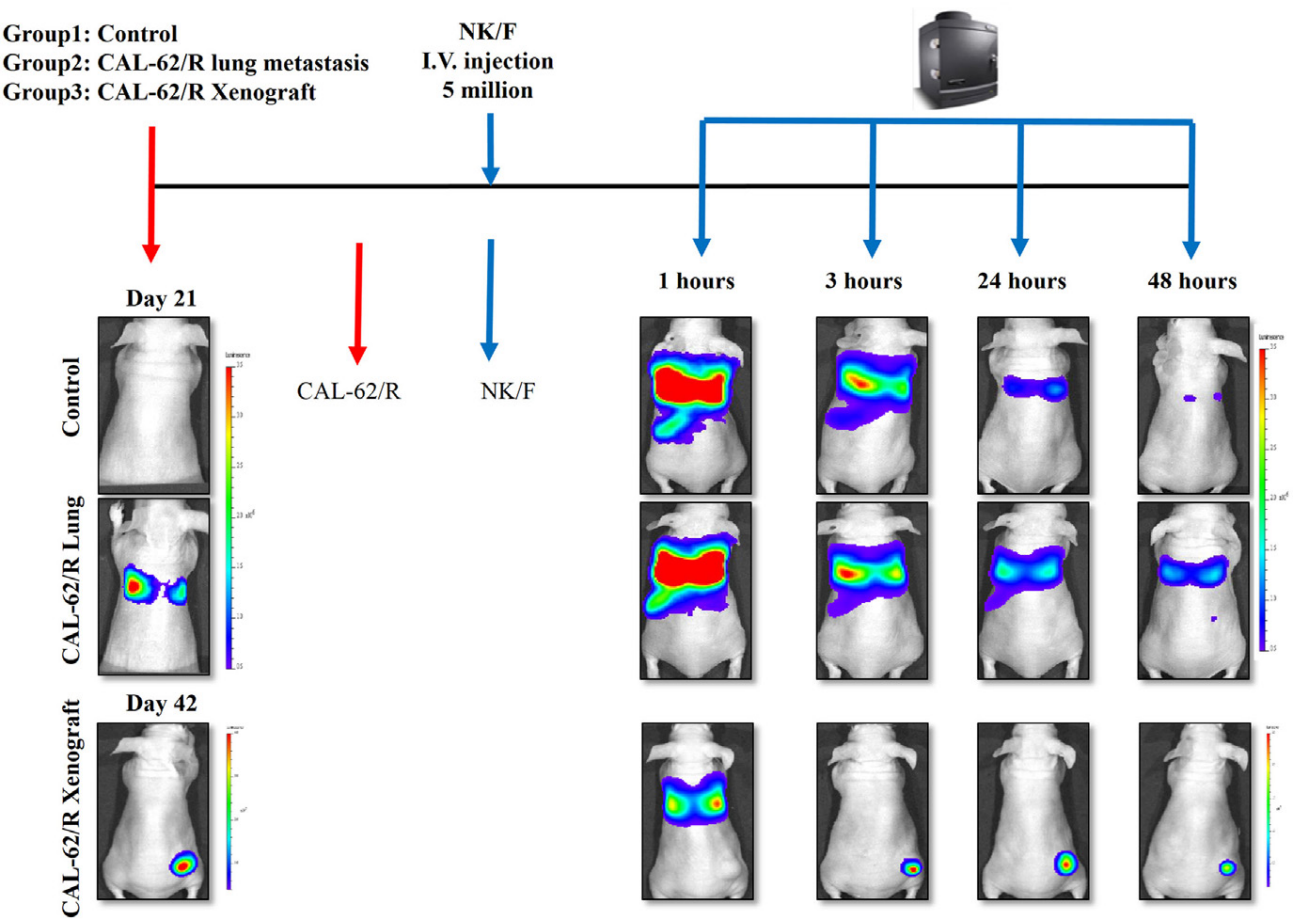
Targeting of NK cells to anaplastic thyroid cancer in animal models with pulmonary metastases or xenografts. CAL-62/R cells (1 × 10^6^ cells/100 μl PBS) were injected into mice *via* the tail vein or subcutaneously in the right thigh. After 21 or 42 days, NK/F cells were intravenously injected. Bioluminescence imaging was measured 1, 3, 24, and 48 h after injection of the NK/F cells. effluc, enhanced firefly luciferase; Rluc, Renilla luciferase; CAL-62/R, CAL-62/Rluc; NK/F, NK-92MI/effluc cells.

The experimental scheme for the *in vivo* bio-distribution of NK cells is summarized in Figure S3 in Supplementary Material. The mice were separated randomly into three groups: the control group, pulmonary metastasis group, and xenograft group. After confirming the tumor conditions by Rluc imaging, five million NK/F cells were injected in the mice *via* the tail vein. Imaging of the NK/F cells was carried out at 1, 3, 24, and 48 h postinjection for each group (Figure 6). The majority of NK/F cells were grouped in the lung and spleen in the pulmonary metastasis group at 1 h postinjection. The BLI signal intensity in the pulmonary metastasis group was almost the same as in the control group (4.38 × 10^8^ vs. 3.87 × 10^8^) at 3 h; however, at 24 h, the BLI signal intensity in the pulmonary metastasis group was 5.79-fold higher than in the control group (1.39 × 10^7^ vs. 2.41 × 10^6^). Moreover, the NK/F cells in the pulmonary metastasis group remained in the lungs longer. For the xenograft group, the bio-distribution of NK/F cells was similar to that of the pulmonary metastasis group at 1 h. However, the NK/F cells gradually migrated to the tumor xenograft area and reached their maximum value at 24 h.

We also studied the retention of NK cells after they had been localized to lung metastases. The NK/F cells were monitored with IVIS imaging for 72 h. The results of this study were in accordance with the results obtained in study 1 (Figure S2 in Supplementary Material). In summary, NK cells showed concordant biological behaviors in both tumor formation and persistence.

### Antitumor Mechanism of NK Cells

**Figure 7:**
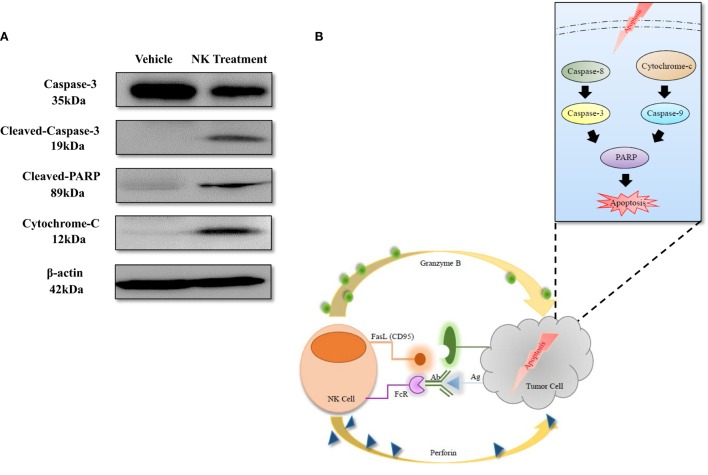
Apoptosis mechanism of anaplastic thyroid cancer induced by NK cell-based immunotherapy. **(A)** Western blotting of proteins related to the apoptosis signaling pathway. **(B)** Diagram of the apoptosis signaling pathway that was induced by NK cells.

To explore the mechanism of NK-92MI cell cytotoxicity against ATC, we selected several key proteins that can reflect the apoptosis signaling pathway in cells: caspase-3, cleaved-caspase-3, cleaved PARP, and cytochrome-C. We also used β-actin as a standard marker. After coculture of CAL-62/F cells with NK cells for 24 h, the amount of cleaved-caspase-3 increased 3.95-fold and the amount of caspase-3 increased 0.59-fold as compared to the control group. The amount of cytochrome-C also increased 2.66-fold, which corresponded with the result for cleaved-caspase-3. As shown in Figure 7A, the amount of cleaved PARP showed a more significant increase (5.83-fold) compared to the control group than the amount of caspase-3 and cytochrome-C. This indicates that the effect on the cleaved PARP was similar to the combined effect on caspase-3 and cytochrome-C.

## Discussion

In the current study, we aimed to develop an NK cell-based immunotherapy for pulmonary metastasis of ATC using molecular imaging technology. To achieve this, ATC and NK-92MI cells expressing a reporter gene were established (CAL-62/R, CAL-62/F, and NK/F). Human ATC cells labeled with the reporter gene emitted a high BLI signal and could be easily detected both *in vitro* and *in vivo* using an optical imaging instrument. Previous studies have used the intracardiac injection of cancer cells to develop a metastasis model (24). Although the injection can result in the widespread dissemination of tumor cells throughout the body, the success rate for establishing metastasis is low. In the current study, intravenous injection of ATC cells labeled with a reporter gene produced pulmonary metastasis of ATC with a low fatality rate in nude mice, and the progression of ATC metastasis was easily monitored with BLI.

In addition, NK-92MI cells labeled with a reporter gene could also be clearly visualized both *in vitro* and *in vivo* using BLI, and the bio-distribution and migration of NK-92MI cells in nude mice with ATC pulmonary metastases could also be successfully monitored using dual reporter gene imaging, demonstrating the simultaneous non-invasive visualization of two types of cells in a single animal. Using the dual reporter gene imaging platform, we demonstrated that the NK cell-based immunotherapy had a satisfactory therapeutic effect on the pulmonary metastases of ATC.

Anaplastic thyroid cancer is rare, but it is one of the most aggressive types of human malignancies (25). There has previously been no effective therapeutic approach for the pulmonary metastases of ATC because of their resistance to conventional therapies (2, 10). In the current study, NK-92MI cells displayed obvious cytotoxicity against ATC both *in vitro* and *in vivo*, which indicates that NK cell-based immunotherapy is a promising therapeutic approach to ATC.

A major challenge in the application of NK cell-based immunotherapy to treat ATC lies in the large-scale production of NK cells. NK cells can be gathered from two main sources for use in immunotherapy: the patient (autologous setting) and a healthy donor (allogeneic setting) (26). It is technically challenging to gather a sufficient amount of active NK cells from the blood of patients because these cells only make up 10% of lymphocytes. The issue of graft-versus-host reactions is another challenging obstacle that needs to be overcome when NK cells are obtained from a healthy donor (27, 28). In the current study, we used the human NK cell line NK-92MI. The NK-92 cell line is the only cell line that has been used clinically and confirmed to be safe with no severe side effects (29–31). However, NK-92 cells are highly dependent on the cytokine interleukin-2 (IL-2), which has toxicity and may cause capillary leak syndrome (6). NK-92MI cells are not dependent on IL-2, and they have been shown to have an antitumor effect on breast cancer cells *in vivo* (3, 4). As shown in Figure 5, NK-92MI cells have a therapeutic effect on ATC.

The mechanisms of NK cell cytotoxicity have been studied previously. FAS ligand (FasL) or tumor necrosis factor-related apoptosis inducing ligand (TRAIL) activates caspase-8 and caspase-3, which leads to the extrinsic pathway of apoptosis in target cells. The combination of perforin and granzyme B leads to the intrinsic pathway of apoptosis, induces the release of cytochrome-c, and finally activates caspase-3, caspase-6, and caspase-7. Both of the apoptosis signaling pathways activate PARP and result in the execution of cell death (5, 26, 32, 33). The results of our study are consistent with those of previous studies; after coculture of NK-92MI and ATC cells for 24 h, cleaved-caspase-3 and cytochrome-c increased, and the level of cleaved PARP also increased significantly (Figure 7), demonstrating that NK-92MI cell treatment induced apoptosis signaling pathways in the cancer cells. This result could explain the therapeutic effect of NK-92MI cells on ATC in both *in vitro* and *in vivo* studies.

Although the effect of NK-92MI cell-based immunotherapy on ATC cells has been shown *in vitro*, the tumor microenvironment could decrease the antitumor effect of NK cells by reducing the release of perforin/granzymes from NK cells and inhibiting Fas/FasL interaction (34, 35); therefore, *in vivo* studies might be more important than *in vitro* experiments to clearly demonstrate the therapeutic effect of NK cells on cancer. Animal models with tumors are usually generated by subcutaneous injection of tumor cells, and the growth or regression of the tumors is monitored by calipers or ultrasound imaging (36, 37). It is usually very difficult to produce an animal model with pulmonary metastases. Previous studies have reported some spontaneous pulmonary metastases; however, they have major drawbacks. These animal models have a low metastasis rate, and the metastases can only be detected postmortem but not in the living model (38, 39). In the current study, we established a pulmonary metastasis model with dynamic tumor development, as shown in Figure 3. Some of the mice also developed bone metastases at a later stage of the disease, which is consist with the clinical situation (2).

The bio-distribution of NK cells may facilitate the generation of an optimal *in vivo* therapeutic protocol. Currently, optical imaging using fluorescence, bioluminescence, or radionuclide imaging with PET or SPECT is applied for this purpose (3, 15, 40). Although PET and SPECT can be applied in the clinics, they have several limitations for their utilization in preclinical studies, such as a high cost, long scan times, a poor spatial resolution, and the lack of suitability for use in long-term imaging due to the decay of radionuclides (11). A previous study had attempted to monitor NK cells in athymic rats *in vivo* by labeling the cells with the near-infrared dye DiD (41). However, the level of labeling can differ depending on the experimental conditions, and the labeling does not permit long-term imaging. In the current study, we inserted a reporter gene into NK-92MI cells, and the labeled cells could be monitored until their death in living subjects. Using optical molecular imaging, we demonstrated the targeting of NK cells to ATC. In the xenograft animal models, NK-92MI cells accumulated in the ATC tumors within 3 h and remained there for more than 24 h. These data are consistent with a previous report by Galli et al. They used an anti-CD56 monoclonal antibody labeled with Tc-99m pertechnetate to show that NK cells targeted the tumor and emerged in the tumor area within 3 h (12). More recently, different methods have been used to enhance tumor targeting by NK cells, such as genetic modification, the use of cytokines, hematopoietic growth factors, and monoclonal antibodies (42, 43).

One limitation of the current study is that only one type of NK cell, NK-92MI, was used in our experiments. The therapeutic effect of human NK cells isolated from healthy donors or patients with thyroid cancer needs to be established for the clinical translation of NK cell therapy in thyroid cancer.

In the current study, an optical imaging platform with the labeling of NK-92MI cells with a reporter gene was established, and this platform can be used to monitor the long-term bio-distribution of NK cells in *in vivo* animal models. This imaging platform can also be used to assess the effectiveness of the different strategies used to enhance tumor targeting by NK cells, and it might contribute to the development of a more successful NK cell-based immunotherapy for patients in the near future.

## Conclusion

In the current study, we developed a dual reporter gene imaging system that can be used to visualize the tumor targeting of ATC pulmonary metastases over time, and monitor the bio-distribution of therapeutic NK-92MI cells in the same animal. The system was used to demonstrate that NK cell-based immunotherapy is a good therapeutic approach for treating ATC pulmonary metastasis.

## Ethics Statement

All animal experiment protocols were conducted in accordance with the National Institutes of Health guidelines for the care and use of laboratory animals and approved by the Committee for the Handing and Use of Animals of Kyungpook National University.

## Author Contributions

Conception and design; drafting of the manuscript: LZ, XJL, and B-CA; execution of experiments: LZ, XJL, HWL, JMO, and SHB; acquisition of data: LZ, XJL, and HWL; analysis and interpretation of data: LZ, XJL, SK, PG, and B-CA; critical revision of the manuscript; final approval of the version to be published: SYJ, S-WL, JL, and B-CA; obtained funding; study supervision; and agreement to be accountable for all aspects of the work: B-CA.

## Conflict of Interest Statement

The authors declare that the research was conducted in the absence of any commercial or financial relationships that could be construed as a potential conflict of interest.
