# Supplementary figures and images for: Natural Killer Cell (NK-92MI)-Based Therapy for Pulmonary Metastasis of Anaplastic Thyroid Cancer in a Nude Mouse Model
Source: Front Immunol. 2017 Jul 21;8:816. doi: 10.3389/fimmu.2017.00816 (PMC5519537; doi:10.3389/fimmu.2017.00816)

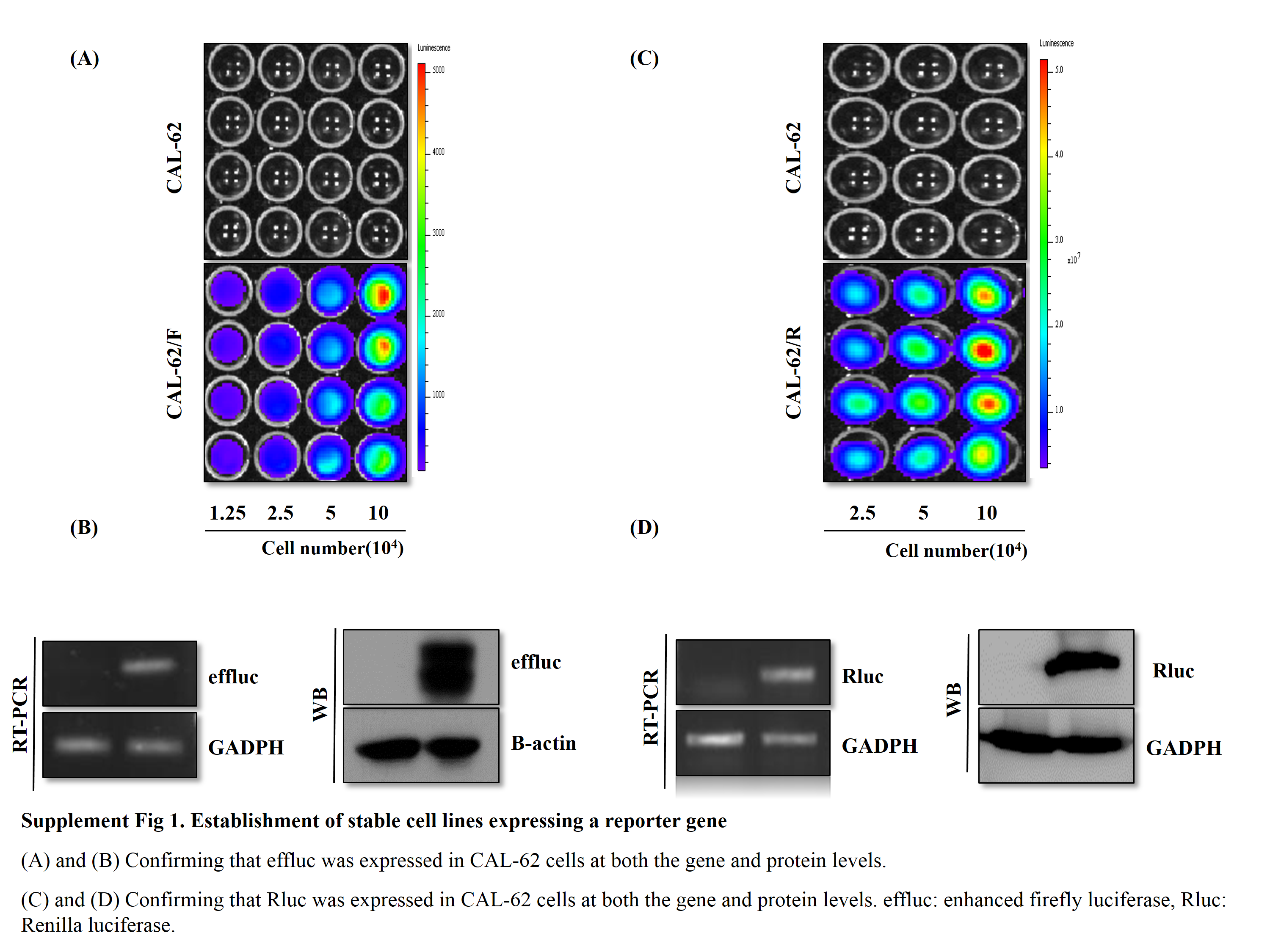

Supplement: Supplementary file 1 [file Image_1.TIF]

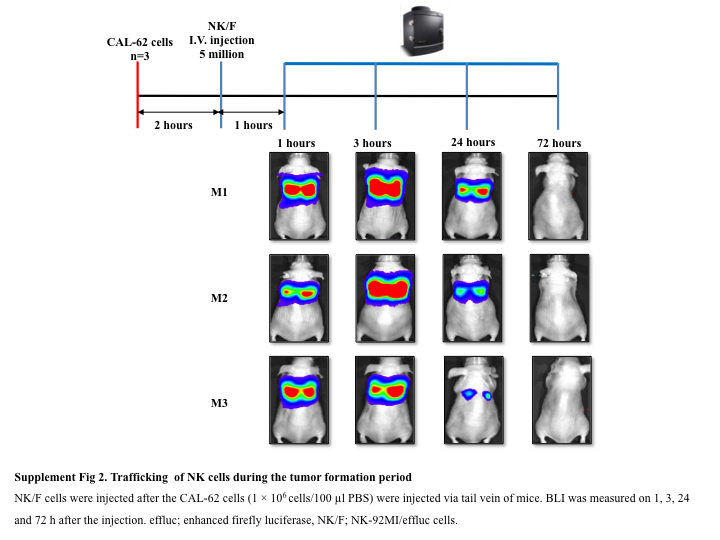

Supplement: Supplementary file 2 [file Image_2.TIFF]

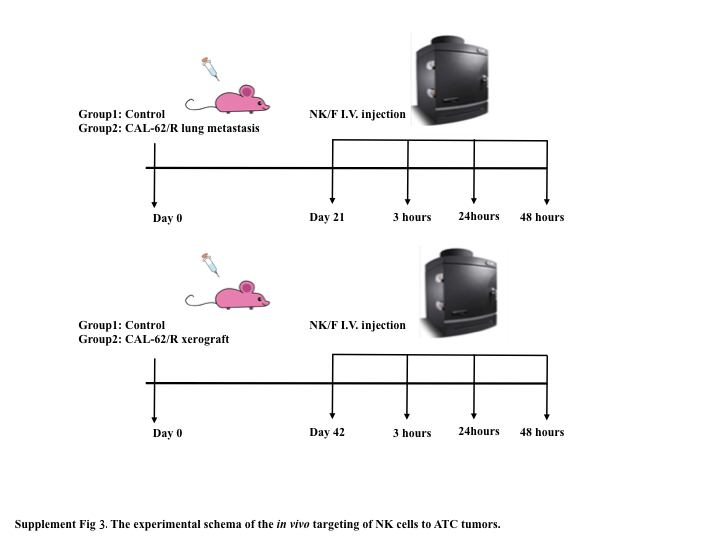

Supplement: Supplementary file 3 [file Image_3.TIFF]

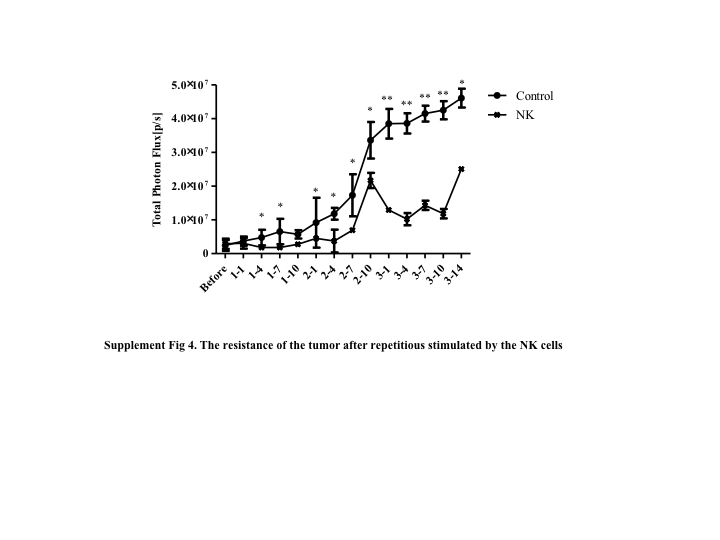

Supplement: Supplementary file 4 [file Image_4.TIFF]
